# Living with tuberculosis: a qualitative study of patients’ experiences with disease and treatment

**DOI:** 10.1186/s12889-022-14115-7

**Published:** 2022-09-10

**Authors:** Juliet Addo, Dave Pearce, Marilyn Metcalf, Courtney Lundquist, Gillian Thomas, David Barros-Aguirre, Gavin C. K. W. Koh, Mike Strange

**Affiliations:** 1 Global Health Catalyst, Global Health R&D, GSK, 980 Great West Road, Brentford, Middlesex TW8 9GS United Kingdom; 2 Product Development and Supply, GSK, Stevenage, United Kingdom; 3 Global Medical, GSK, Research Triangle Park, USA; 4 Adelphi Research, Bollington, United Kingdom; 5 Global Health Pharma Research Unit, Tres Cantos, Spain; 6 Global Health Clinical, Stockley Park West, United Kingdom

**Keywords:** Tuberculosis, Treatment failure, Adherence, Stigma, COVID-19

## Abstract

**Background:**

Although tuberculosis (TB) is a curable disease, treatment is complex and prolonged, requiring considerable commitment from patients. This study aimed to understand the common perspectives of TB patients across Brazil, Russia, India, China, and South Africa throughout their disease journey, including the emotional, psychological, and practical challenges that patients and their families face.

**Methods:**

This qualitative market research study was conducted between July 2020 and February 2021. Eight TB patients from each country (*n* = 40) completed health questionnaires, video/telephone interviews, and diaries regarding their experiences of TB. Additionally, 52 household members were interviewed. Patients at different stages of their TB treatment journey, from a range of socioeconomic groups, with or without TB risk factors were sought. Anonymized data underwent triangulation and thematic analysis by iterative coding of statements.

**Results:**

The sample included 23 men and 17 women aged 13–60 years old, with risk factors for TB reported by 23/40 patients. Although patients were from different countries and cultural backgrounds, experiencing diverse health system contexts, five themes emerged as common across the sample. 1) Economic hardship from loss of income and medical/travel expenses. 2) Widespread stigma, delaying presentation and deeply affecting patients’ emotional wellbeing. 3) TB and HIV co-infection was particularly challenging, but increased TB awareness and accelerated diagnosis. 4) Disruption to family life strained relationships and increased patients’ feelings of isolation and loneliness. 5) The COVID-19 pandemic made it easier for TB patients to keep their condition private, but disrupted access to services.

**Conclusions:**

Despite disparate cultural, socio-economic, and systemic contexts across countries, TB patients experience common challenges. A robust examination of the needs of individual patients and their families is required to improve the patient experience, encourage adherence, and promote cure, given the limitations of current treatment.

**Supplementary Information:**

The online version contains supplementary material available at 10.1186/s12889-022-14115-7.

## Background

Tuberculosis (TB) is a communicable infectious disease affecting around one quarter of the world’s population [1]. The ‘BRICS’ countries of Brazil​, Russia, India, China, and South Africa account for 47% of the total number of TB cases annually [1–3].

Caused by the bacillus *Mycobacterium tuberculosis*, around 5–10% of those infected will develop active disease. In 2019, 10 million new active cases and 1.4 million deaths were reported [1]. In 2020, the coronavirus disease 2019 (COVID-19) pandemic severely impacted the reporting of new cases and impeded diagnosis and treatment [3]. Treatment for multidrug-resistant TB (MDR-TB) also declined by 15% (from 177,100 in 2019 to 150,359 in 2020), with only about a third of patients who needed this treatment obtaining access [3].

Ambitious targets to end the TB epidemic by 2035 were established in 2015 by the WHO’s End TB Strategy [4], aligned with the United Nations Sustainable Development Goals [5]. In 2018, a United Nations General Assembly High-Level Meeting on Tuberculosis resulted in a Political Declaration on Tuberculosis, committing to end TB globally by 2030 [6]. Achieving these goals requires more equitable deployment of existing measures, and the development of new tools for TB prevention, diagnosis and treatment [7]. Progress towards ending TB also demands that interventions are aligned to patients’ experiences and address the challenges that they face [8, 9].

TB typically involves the lungs (pulmonary TB) and is acquired via inhalation of droplet nuclei in the air following exposure usually over several hours. Close contact and the infectiousness of the source patient are key risk factors for the infection of tuberculin-negative persons [10]. Current treatment of drug-susceptible TB requires combination therapy consisting of an intensive phase of 2 months of isoniazid, rifampin, pyrazinamide, and ethambutol, followed by a continuation phase of 4 months of isoniazid and rifampin [11]. Directly observed therapy (DOT) is recommended to ensure adherence to the complex regimen and to deter the emergence and spread of MDR-TB. Treatment is successful in around 85% of patients following 6 months’ therapy [1]. Also, individuals can become non-infectious within two weeks of treatment initiation, restraining disease transmission [1]. Thus, prompt initiation of therapy is important for both the patient and their close contacts. However, the management of TB is complicated by the increasing prevalence of MDR-TB, which requires prolonged and complex therapy, and is more likely to be associated with poor outcomes [12]. Even after successful treatment, patients may have ongoing lung disease and a decreased life expectancy [13–15].

The drugs used to treat tuberculosis are well understood clinically, and susceptibility testing will indicate which treatment regimen is appropriate [11, 12]. However, treatment effectiveness depends on patient adherence to a demanding and lengthy treatment regimen with associated side effects. In this context, a patient-focused approach which considers the individual’s specific circumstances is needed to ensure sufficient adherence and good outcomes from therapy. Interest in this field has been building steadily and is most suited to a qualitative investigational approach which allows deep exploration of motivations, reactions, goals, aspirations, and circumstances. However, studies more often consider the challenges faced by healthcare workers caring for TB patients [16], or the implementation of new management tools [17, 18].

Previous studies have examined how patients manage their illness and the impact that TB has on their daily lives, their families, and the wider community [19, 20], as well as the stigma associated with poverty and HIV and the effects of discrimination [21]. However, defining studies on the experiences of TB patients and their families are not available for all the BRICS countries, and comparison between studies with different methodologies and objectives is problematic. It is, therefore, unclear to what extent the experiences of TB patients are shared across countries.

We report the findings of a qualitative evaluation of TB patients’ experiences across the five BRICS countries. The study aimed to identify commonalities across the different country contexts, by examining the perspectives of TB patients throughout their full disease journey, including the emotional, psychosocial and practical challenges that patients and their families face. A greater understanding of these factors could inform care more focused on patients’ needs, with the aim of improving outcomes and directing the development of new tools to end TB.

## Methods

### Study design

This qualitative market research study was designed collaboratively by GSK and Adelphi Research and conducted between July 2020 and February 2021 across the five BRICS countries (Brazil, Russia, India, China, and South Africa). The study was non-interventional and without clinical endpoints. The aim was to achieve a better understanding of the TB market across the BRICS countries by identifying common challenges faced by TB patients and their families in their daily lives throughout their treatment journey.

The study conformed to ethical principles laid down in the Declaration of Helsinki, all national data protection laws and industry guidelines. Participants’ data was protected by compliance with General Data Protection Regulation [22]. All participating patients and household members provided written voluntary informed consent, and parents provided written consent for children under the age of consent. Consent was also provided for anonymized publication of the findings. For consent forms see supplementary materials, Additional file 1.

To investigate the experiences, meanings, and perspectives of TB patients, qualitative methodology was employed to identify themes within and across countries from in-depth interviews and self-recorded videos, supported by a self-completed health questionnaire.

Participants with experiences relevant to the study objectives were actively recruited from BRICS countries because they account for more TB cases than any other country in their respective WHO regions, and because of the different additional challenges confronting these countries such as the burden of TB-HIV co-infection in South Africa, the diversity of private sector care in India, and the burden of MDR-TB in India, China and Russia [1, 2, 23]. Remote data collection both preserved the privacy of participants and ensured the safety of moderators given the infectious nature of TB and the timing of the study during the COVID-19 pandemic.

### Recruitment

Participants were recruited through independent healthcare fieldwork agencies in the different countries via referral from healthcare professionals and social or community workers, as well as using market research databases, posters and adverts in TB clinics, patient groups, and word of mouth referrals. Participants had the opportunity to discuss the study with recruiters before completing a screening guide to confirm patient eligibility (Additional file 2). Recruited participants received an honorarium at fair market value for their participation.

Recruitment continued until TB patients from 40 households, that is 8 per country, plus 1–5 members of their households had been sampled. The minimum target sample size was 80 participants. Previous studies have indicated that for this type of qualitative research as few as 6 interviews per setting are required to identify major themes [24, 25], with saturation occurring within 12 interviews [26].

### Participants

Eligible participants had a confirmed diagnosis of TB and were receiving treatment or had completed treatment within the previous 12 months. Close family and other household members were included where appropriate for support and additional information, except for China where the social stigma prevented discussion with individuals other than the patient. Participants were recruited from a range of socio-economic backgrounds, assessed based on income, education levels, and living standard. At least three participants from each country were to be female. The study sought to include a range of specific patient types, for example, persons living with HIV (PLWH), those with diabetes, smokers, those with a history of excessive alcohol consumption, and those with MDR-TB/relapsed TB. At least two patients per country were to be living in households which included a child diagnosed with TB or receiving preventive treatment. No participant was excluded because of lack of access to technology as the necessary equipment was loaned to participants where needed.

### Data collection

The interview moderators, fluent in the local languages, were taken through a training process in each setting, detailing study objectives, inclusion criteria, and study methodology, followed by subsequent monitoring of the process and active feedback to ensure quality control. Data quality was assured by consistent and thorough briefing of the field workers, including regular follow up to ensure study procedures were followed. The discussion guide and videoing instructions were carefully designed to contain clear respondent instructions at each question.

Patients first completed a 5-min health questionnaire based on their physical health over the previous four weeks. Interviews with TB patients and household members were conducted remotely by a trained moderator in the form of either a 60-min video-streamed interview or a 60-min telephone interview. The questionnaire and interview guides are provided in the supplementary materials (Additional file 3). Participants also completed a 45-min follow up video task to create four short videos on a mobile phone in their own time to capture their personal experience, such as their living environment, changes in their living arrangements as a result of TB, the biggest challenges since the diagnosis, perception of the changes in their life from others around them, and their hopes and expectations for the future.

### Analysis

The interviews were transcribed verbatim from the original languages, that is: Brazil, Portuguese; Russia, Russian; India, Hindi and English; China, Mandarin; South Africa, English, Sesotho, isiZulu, Tswana, or Afrikaans with switching between languages as necessary. Following translation into English, the information was analysed manually using a thematic and comparative analysis approach to identify key themes both within countries and across all participants’ responses [27, 28]. Analysts had no access to patient medical records and all patient identifying information was anonymized.

Interviews were coded thematically by three analysts, aiming to reach consensus through regular team meetings where the emerging findings were discussed. Additionally, non-verbal communication (including visual evidence of living conditions) present in the videos from the streamed interviews and the video tasks were shared with the full team at regular intervals and discussed/analysed using the thematic framework developed from the transcripts. Triangulation across the different data sources was done using cross-checking to assess convergence, complementarity and divergence at the individual participant level, between patients and their families, and at the country level between informants from the same country. The analysis was therefore grouped initially by country and then analysed for cross-cutting themes across all respondents. Quality control was achieved by continuous review by two senior analysts, one of whom was not involved in the initial analysis, plus a final check through all the analyses.

## Results

### Participants

**Fig. 1:**
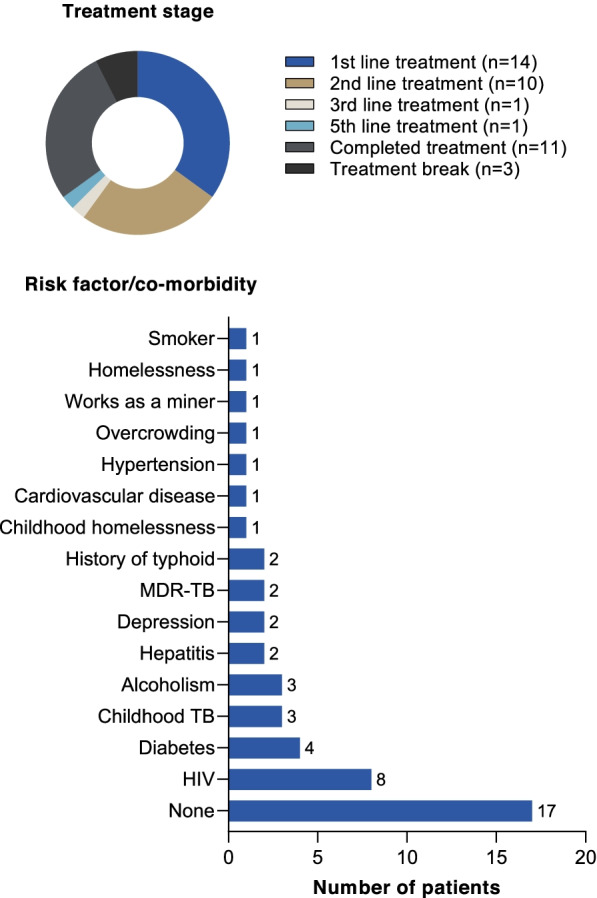
Summary of patient characteristics. Note that patients may have had more than one risk factor/co-morbidity

The sample consisted of 40 TB patients (8 from each country) plus 52 household members. Each patient was assigned an identifier to illustrate their country and number. Of the TB patients, 23 were men and 17 women, ranging between 13 and 60 years old. Fourteen were receiving first-line treatment, 10 second-line treatment, 2 patients had received multiple treatment lines, 11 had completed treatment, and 3 patients (all from Russia) were on a treatment break (Table 1, Fig. 1). Risk factors for TB were reported in 23/40 patients, with some patients having multiple risk factors (Table 1, Fig. 1). Most patients were of medium socio-economic status for their country (26/40), and no patients with high socio-economic status were recruited (Table 1). Except for India and South Africa, it was not possible to recruit at least two households with a child diagnosed with TB or receiving preventive treatment (Table 1).

**Table 1:** Characteristics of TB patients participating in the study by country

Characteristic	Brazil	Russia	India	China	South Africa
Self-reported gender					
Female	5	4	3	3	4
Male	3	4	5	5	4
Healthcare coverage					
Public	8	8	6	7	7
Private	0	0	2	0	1
Both	0	0	0	1	0
Socio-economic status^a^					
High	0	0	0	0	0
Medium	6	4	6	6	4
Low	2	4	2	2	2
Not provided	0	0	0	0	2
TB treatment status					
1^st^ line	4	1	3	2	4
2^nd^ line or more	2	4	1	5	0
Treatment complete	2	0	4	1	4
Treatment break	0	3	0	0	0
Risk factors^b^					
None	4	1	3	6	3
HIV	1	3	0	0	4
Diabetes	1	1	1	0	1
Childhood TB	2	0	1	0	0
Depression	0	2	0	0	0
MDR-TB	0	2	0	0	0
History of typhoid	0	0	2	0	0
Childhood homelessness	1	0	0	0	0
CV disease	0	1	0	0	0
Alcoholism	0	2	0	0	1
Hepatitis	0	1	0	1	0
Hypertension	0	0	1	0	0
Overcrowding	0	0	1	0	0
Works as a miner	0	0	0	0	1
Homelessness	0	0	0	0	1
Smoker	0	0	0	1	0
Number of family participants	6	6	19	0	21
Households with ≥ 1child:	2	4	5	4	4
No preventive medication	0	0	2	4	1
Preventive medication	0	1	2	0	2
Previous preventive medication	0	0	0	0	1
TB diagnosis for child	0	0	1	0	0
Child status not disclosed	2	3	0	0	0

^a^Relative within the individual country, including income and educational measures

^b^Participants may have had more than one risk factor

### Patient health status

**Fig. 2:**
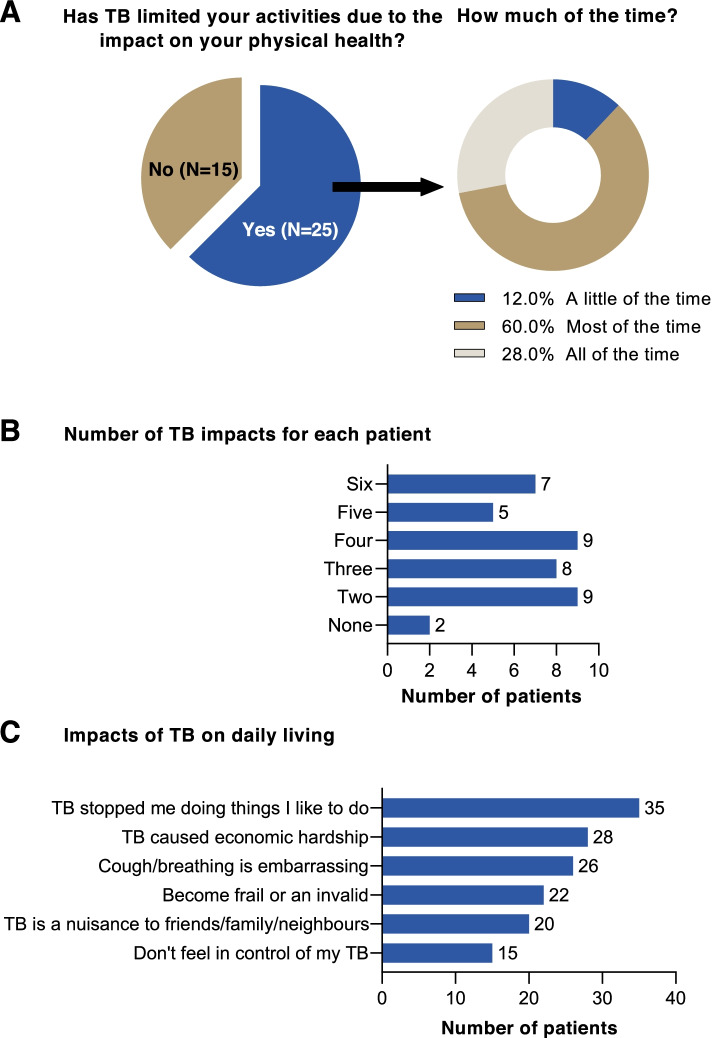
Results of a self-reported health questionnaire. **A** The effect of TB on limiting daily activity due to patients’ physical health; **B**) the impact of TB on daily living; and **C**) the number of impacts on daily living experienced by patients

The self-reported health questionnaire indicated that most respondents (25/40) found that the physical impact of TB limited their activity. A higher proportion of patients who were currently receiving treatment (69.6% [16/23]) reported a physical impact of TB compared with those that had completed treatment (57.1% [8/14]) or who were on a treatment break (33.3% [1/3]). Most patients whose physical activity was impacted by TB reported that this affected them all or most of the time (88.0% [22/25]) (Fig. 2A). Most patients (38/40) reported that their daily living was impacted in at least two ways (Fig. 2B). Seven patients, five of whom were receiving treatment and two who had completed first-line treatment, stated that they were impacted by all six areas assessed (Fig. 2B). Looking at specific impacts, the most reported were that TB stopped patients doing things that they liked to do (35/40), and economic hardship (28/40) (Fig. 2C). Overall, it was clear that TB had significantly impaired the health status of patients and had a negative impact on daily living.

### Patient journey

#### Pre-diagnosis

The most common initial symptoms reported by patients were a long-lasting cough increasing in severity over time, fever, weight loss, and tiredness. Some patients experienced more severe symptoms such as haemoptysis, and pleural effusion. However, symptoms were often non-specific, and unless they were aware of a source of infection or had known risk factors (e.g. HIV), most patients did not consider TB as a potential cause. Notably, patients in South Africa were more likely to suspect TB because of a higher awareness in the community and the link with HIV. In India, recent typhoid infection was suspected as the cause of symptoms in some cases.

Patients tended to hope that the symptoms would resolve on their own using over-the-counter products and traditional medicine. Patients with addiction to alcohol did not always perceive the severity of their symptoms and were less willing to engage with healthcare providers. However, avoidance of healthcare providers was common across all settings, because of concerns for the associated costs.

“The symptoms were there for the last 2 ½ months but I did not know. He was coughing a lot, so I asked him to go to the doctor. He did not listen to me. He feared talking to the doctor.” Relative of TB patient, India (IN19).

“One day, I started to have fever in the afternoon. After work, I went to receive infusion in a small local clinic. I remember my body temperature was 39.5 to 39.6 degrees Celsius. The doctor said my condition was very serious, so he prescribed 5 bottles of infusion to me, and I received all of them. But my fever persisted after such a lengthy infusion.” China (CN09).

#### Diagnosis

**Fig. 3:**
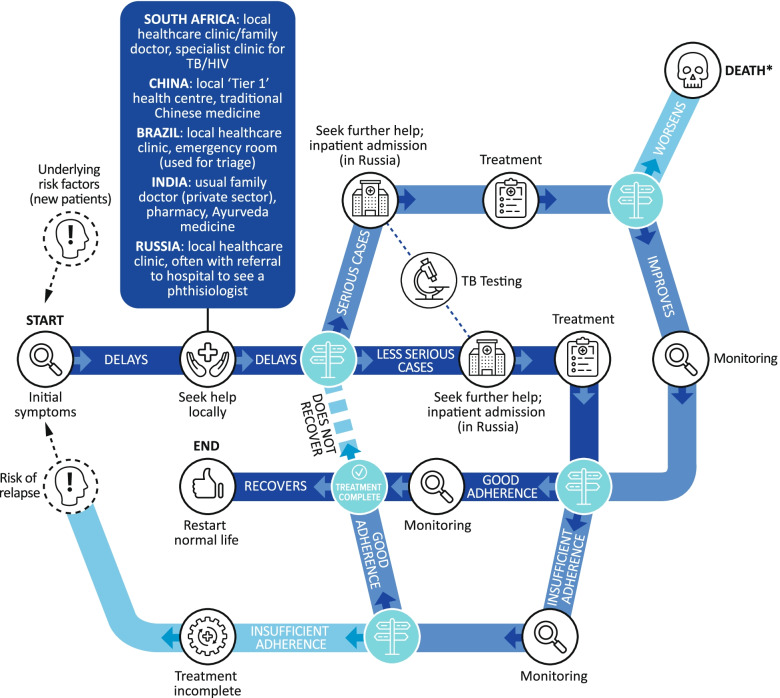
The TB patient pathway. *There were no deaths during the study

The pathway for TB cases depended on symptom severity at presentation but navigating the healthcare system was tortuous for some patients. Patients first sought help using a familiar and accessible route (Fig. 3).

Across all countries, the TB diagnosis came as a shock to most patients – their initial thought was ‘Will I die?’. PLWH were less surprised as they were aware of the association with TB. Some patients in South Africa believed they had been vaccinated against TB as children and were therefore protected. Many patients questioned how they had caught TB and worried about the negative misconceptions associated with the disease, particularly in Russia and Brazil. Patients feared that they would be ostracized and shunned by their families and communities. Young people with TB feared for their future, for example their careers, education, and prospects of marriage. Further concerns expressed by patients included the potential disruption to their life, job security and providing for their dependents, especially in India. Overall, there was uncertainty among patients as to whether they could cope; some expressed the fear of unintentional disclosure of their TB diagnosis to others. Notably, across all countries, families were often fearful of the potential costs, with a lack of clarity regarding which elements of treatment would be covered by insurance (where available) or were refundable from the public health system.

“[I thought] it is some kind of prison disease, which occurs more and more often in people who have served a sentence somewhere. That is, more disadvantaged groups of the population. I always thought about it in this way until I met it myself.” Russia (RU10).

Following diagnosis, healthcare providers were quick to reassure patients that TB is treatable but that it will take time and that they must try not to infect others. In South Africa some patients reported being warned of drug resistance. However, beyond this, TB-focused education was limited, and patients often conducted their own research via the Internet and word of mouth, though patient-friendly resources were described as inadequate in some settings.

“[The nurse] said if you don’t take your meds, they send you to [a TB hospital] and then you will receive extreme treatment. They inject you with needles and stuff. That is if you don’t use this meds at home, they will send you there and stay for six months.” South Africa (SA05).

#### Treatment

**Fig. 4:**
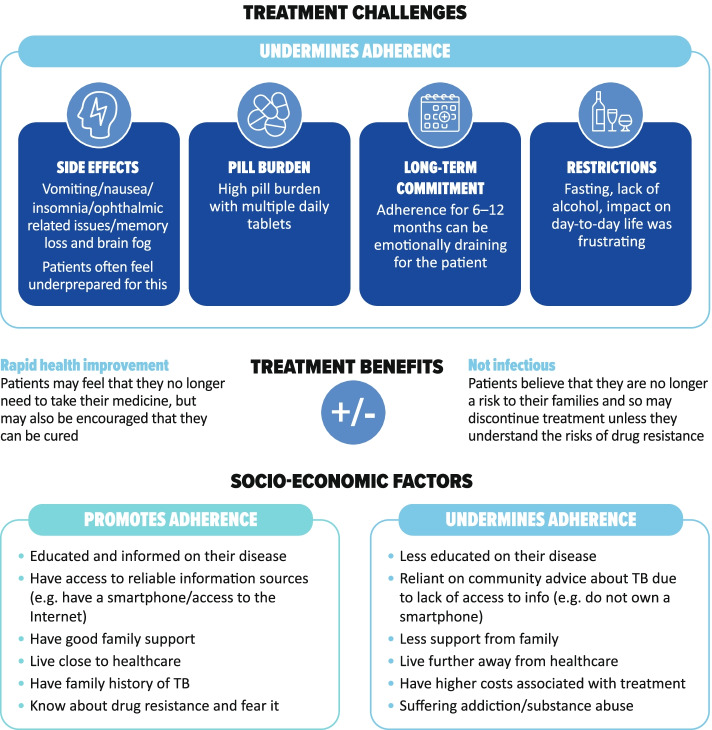
Factors identified by patients as affecting adherence to TB therapy

Treatment side effects, pill burden, lifestyle restrictions and the long-term commitment required were very challenging for patients (Fig. 4). Patients generally did not know the names of their medications, but described having to take many pills of different types several times a day. Patients reported intolerable side effects, including nausea and vomiting, and patients with MDR-TB faced painful daily injections. In Russia, and to a lesser extent in China, patients were admitted to hospital to increase adherence. In Russia, patients recounted being admitted to sanatoriums for the treatment of TB.

“I take many anti-TB pills every day, covering 4–5 classes, about 20 tablets in total. Sometimes, it’s difficult for me to take medication, as I was quite reluctant to take it initially, but I had no choice, but to take it as a treatment.” China (CN11).

#### Monitoring and adherence

Across countries and socioeconomic bands, patients perceived minimal therapy monitoring by healthcare providers, with little evidence of DOT. It is possible that this was because of interruption to normal healthcare services because of the COVID-19 pandemic (see below). Most patients visited healthcare settings frequently to pick up their medications. Less frequently, their weight was measured during clinic visits, sputum tests were conducted, and some patients were informed when they were no longer infectious and could return to work/education. Family played a key role in monitoring during treatment, encouraging patients to continue with their treatment, sharing regular reminders, and helping to pick up medication from health centres. Motivation to comply was prompted by the desire to get back to normal family life and work, the fear of death, potential drug resistance, and hospitalization. Although patients would briefly lapse without serious consequences, they were usually encouraged to continue treatment by family and healthcare providers.

“Sometimes [redacted] forgets to take the medication, and I argue with him because if one of us forgets the treatment and the other one doesn’t then it won’t work, if we don’t take it together, it won’t work.” Brazil (BR04).

Once treatment was initiated, health improvements were quickly apparent to most patients, with resolution of fever and abatement in cough. Although this increased patients’ optimism and secured a return to some of their previous activities, it could also lead patients to believe that they had recovered, undermining adherence to therapy. Adherence was also jeopardized where there were high barriers to accessing treatment, a poor understanding of drug resistance, and when patients were alcohol dependent (Fig. 4). Patients who did adhere to treatment were often well supported by family and well informed of the consequences of non-adherence. Conversely, those who did not adhere to treatment were often unaware of the consequences.

“By December I was already feeling like I’m already cured, I nearly decided not to continue with the treatment.” South Africa (SA01).

“Actually, they didn’t tell me about the details then. It was very important to emphasize it to me, but the physician didn’t do it. If he did, it would draw my attention and it won’t lead to drug resistance, as I often missed the dose I was supposed to take.” China (CN06).

“I live in a little town which is quite far from the city. I can either go by bus which takes at least an hour and a half, or I can get to the nearest bullet train, but there aren’t many trains available and they are expensive.” China (CN08).

#### Completion of treatment

Eleven patients had completed treatment, 4 from South Africa, 4 from India, 2 from Brazil, and 1 from China. All had recovered, 10 following first-line treatment and 1 following second-line treatment (India). Some respondents said that their time in isolation was a time of reflection where their lives had been ‘put on pause’ making them ‘appreciate the little things in life’ they had really missed. A few patients said that their experience with TB has driven them to want to increase awareness, and remove stigma around the disease e.g., patients in Brazil and China set up informal support networks with fellow patients, particularly where patients met during hospital stays. Most patients expressed relief that they were cured, and that treatment was over, and were generally hopeful for their future.

"My TB is cured, and I want to start again with my studies. I was preparing for a railway job but I had to give that up because of TB. Now I will start my studies again and apply for a government job." India (IN04).

"Thanks to this [TB] I got rid of bad habits, I do not drink alcohol now and smoke less… And I found a job, and I earn some money at the moment, during the first period my brother supported me fully, thanks to him, and my mother helped what she could.” Russia (RU05).

"After these three months since I have recovered, this is what it has brought me, the willingness to fight, to battle, also to take even more care of my health, not just mine but also of people around me, and take this story, my testament, my lived experience with TB… So it’s a goal in my life, to spread information among all those who are close to me." Relative of TB patient (BR01).

#### Access to services

Before TB was diagnosed, in some cases patients consulted healthcare providers in the private sector, for example, the local family doctor, traditional medicine providers, or pharmacies. Following diagnosis, more affluent patients claimed on insurance or paid for private sector treatment due to poor perceptions of the public sector, and some sought support in the private sector for a ‘second opinion’ or for problems which they felt were not being addressed in the public sector. However, the majority of patients (36/40) obtained their TB care through the public sector; three patients used the private sector with one accessing both public and private sector healthcare. Treatment was provided for free through the national programs, with relatively good access in most settings, though travel distance and wait times were a barrier to access. There were reports of drug stock outs and out of pocket expenses for additional diagnostic tests or prescriptions, including having to pay for MDR-TB treatment in some settings (China). A minority of patients reported being turned away from the public sector for not having the correct paperwork or not being able to book an appointment. The public sector had a poor reputation for long queues and poor service and most patients aspired to be able to afford private treatment where services were described as being better.

“In public [sector healthcare] those nurses don’t care, I remember when I accompanied him, I was told I was not allowed to get inside, so he went in on his own. You go in pick up whatever you need and get out because those people don’t have time for anything.” Relative of TB patient, South Africa (SA01).

“In the Government hospital, the doctors do not listen to us. They come when they wish and give medicines. As it is, the doctors do not listen to poor people. I had to buy some medicines from outside.” Relative of TB patient, India (IN17).

“Obtaining the medication – because the drugs can only be obtained in the hospital, you can’t buy them in retail pharmacies. If I run out of my medication, I wouldn’t be able to buy it from the retail pharmacy, I would have to go the hospital, which is inconvenient.” China (CN08).

The use of sanatoriums in Russia was unique. Following diagnosis, patients were sent to a dedicated facility or a TB unit within a hospital where they remained for at least 3–4 months, though confinement could last for up to a year. They were only allowed to leave with permission, for example, at weekends or holidays. Although patients generally accepted that it was for the ‘greater good’ it was frightening at first because some other patients on the ward had very severe disease. However, some patients expressed surprise that other patients were ‘normal’, because they believed the disease to be often associated with homelessness and prisons.

“They told me I had a resistant form of TB and that the treatment is very, very long lasting. At first, they said I would have to be hospitalized three to four months and that then I would be able to go home but when I got to the hospital, the ‘girls’ told me that three to four months is optimistic… In short, eight months. Eight in the hospital and a year after the hospital. That was a shock.” Russia (RU12).

“In my room there were all young women and all were so great. All of them were socially adapted: an accountant, a paediatrician student. So, let’s say it was good company.” Russia (RU01).

### Thematic analysis

**Fig. 5:**
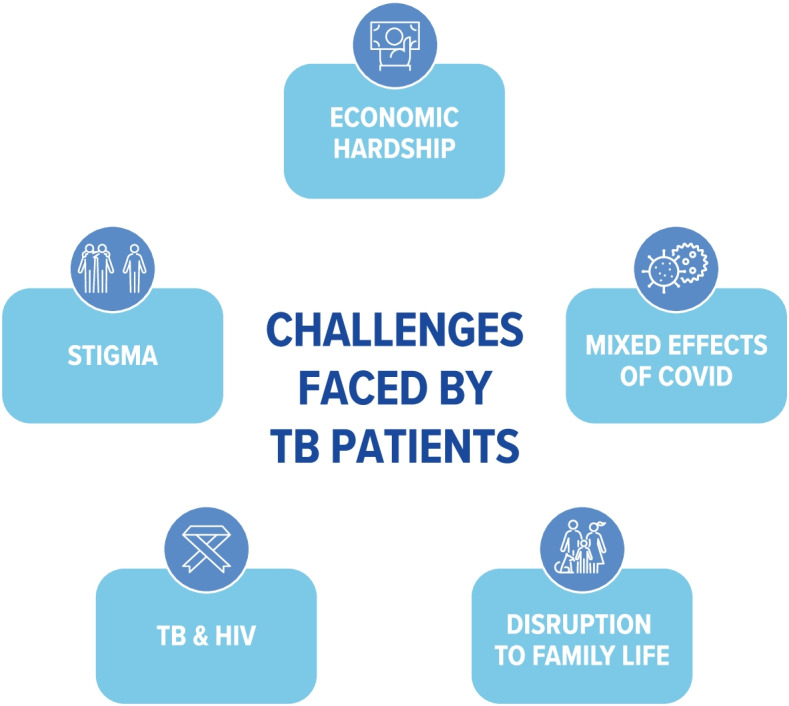
Thematic areas identified as common across five countries describing the challenges faced by TB patients

Five major themes were identified as common across all the countries studied (Fig. 5).

#### Economic hardship

Loss of earnings has the greatest economic impact for TB patients. Most patients stopped work because they felt too unwell to continue or were embarrassed by the symptoms, such as the persistent cough and severe weight loss. Some patients also felt the need to stay away from work to limit transmission to others or were ‘asked to leave’ by their employers as they were not covered by contracts. Many had no entitlement to sick pay. In some cases, patients were concerned that their financial situation could get worse as their diagnosis may mean prospective employers may be reluctant to take them on.

“The main problem is money. There is no problem greater than financial problems.” India (IN01).

“I had to keep away from work because there was a lot of dust involved.” Brazil (BR15).

“I cannot officially get a job, and I cannot unofficially either. But, what? Am I going to work as a loader? I cannot. This has seriously affected my finances… And who would hire if information comes out that there was TB? You will not get a job. I received a disability [payment].” Russia (RU05).

Even in regions where TB treatment was publicly funded, associated costs such as tests, hospitalization, prescriptions, travel, special food/supplements to manage weight loss, and medications to manage adverse effects were often borne by patients. The financial impact of TB meant that most patients had to rely on family or sometimes charities for support or take out loans. Time off for appointments still impacted earnings even after patients had returned to work.

“I also buy medications at my own expense [for gastric side effects] i.e. for TB, everything is free of charge due to the medical insurance policy, everything is fine, but if there is something secondary or something else not related to the diagnosis, then that is at your own expense.” Russia (RU07).

“We are not educated people. I just wanted my child to recover. We are poor people; we could not work during lockdown. We had to borrow money from many people and requested help from doctors too. I thought my child would recover, but he did not. We were very stressed out.” Relative of TB patient, India (IN21).

“To avoid delaying treatment, the doctor told me to take these four drugs upon diagnosis, and urged me to buy them elsewhere, as they were unavailable in the hospital. My wife found they were unavailable in many pharmacies either. Finally, she found them in several pharmacies, from where we bought them in early stage.” China (CN09).

#### Stigma associated with TB

Across all countries stigma was associated with TB, though it manifested in different ways. In China, TB was often kept a secret, even from family, whereas in South Africa, there was greater openness. In Brazil, though patients were open with family, there was reluctance to acknowledge their diagnosis with their community as TB is associated with wider social issues such as poverty, incarceration and ‘immoral lifestyles’. In India, TB patients felt discriminated against for other reasons, such as poverty, as well as TB. Stigma in Russia was related to the personal circumstances of the patient.

Young patients faced bullying at school/college and being dropped by friendship groups. Adults were ostracized by friends and relatives afraid of contracting TB, and relationships with friends and family suffered, leading to loneliness and depression. Respondents described instances when they were not invited to family events even after they had completed treatment and were cured. In some cases, TB appeared to ‘run in families’ meaning the stigma was intergenerational. Importantly, a family with TB was often considered a ‘low status’ family and this was compounded by the financial difficulties that accompany TB.

“A lot of my friends kept away from me because of this, because that’s what people know, that it’s contagious, but they don’t understand that the person on the other side is suffering as well, and we don’t only suffer a little bit, at least myself, it’s a very painful process, very painful, very complicated.” Relative of TB patient, Brazil (BR01).

“The community was no longer as close to us because we are staying with a person that has TB – people at the queue at shops would turn around and come back when we have left.” Relative of TB patient, South Africa (SA14).

“When a person has TB he becomes very annoyed as he has to go through a lot of things, plus there also comes a phase were people start avoiding you, they feel that if we come in contact with this person even we might acquire it.” India (IN01).

“A person who has TB is not somebody who is well-regarded.” Brazil (BR04).

#### TB and HIV

HIV co-infection played a major role in the TB experience, particularly in South Africa. Awareness of TB was higher among PLWH given their greater risk and regular contact with healthcare services. Also, the path to diagnosis was shorter given their engagement with HIV services with rapid referral reflecting the associated co-infection risks. In many cases, the HIV and TB clinics were co-located improving patient access. However, PLWH were highly aware of the stigma that TB carries with fear around the community reaction during the early stages of their journey.

"Now I’m scared I’m HIV positive, I have TB and now there’s Corona [COVID-19], what’s going to happen when I have all three of them?" South Africa (SA18).

“So people were really scared, I think they are now more afraid of TB than HIV. I told my neighbour that I was diagnosed with TB and luckily she doesn’t talk much but still I was aware of their behaviour when they came by to do my laundry they would wait outside to hand it over to them and when they are done they would leave it by the door." South Africa (SA10).

#### Disruption to family life

A diagnosis of TB affects everyone in the household and the wider family. Cleaning and disinfecting routines have to be established and maintained, and there was a general awareness that separate cutlery must be used, living spaces needed good ventilation, and clothes and bedding should be washed more frequently. Sleeping arrangements to isolate TB patients were particularly problematic in India and South Africa where large families live together, and parental co-sleeping with children was no longer possible where this was practiced. In some cases, children were looked after in the homes of extended family members, away from parents with TB. Married patients feared abandonment or divorce and respondents felt ‘lucky’ that their partners had stayed with them despite their TB status. The reduced family contact, demands of treatment and financial hardship often strained family relationships.

“Life at home isn’t the same because I had to begin separating my cutlery and a glass – my clothes had to be washed separately, we have to clean down the house and open the windows to let the air circulate.” Brazil (BR15).

“I’m worried I may infect my parents. So I’ve had to reduce my interactions with them, the time spent with them, the number of occasions I’m with them. And as they get older, they become confused and they don’t understand why I stay away.” China (CN08).

“Our house always used to be full at weekends, friends would come around to watch films, sometimes we would make lunch, get pizza and sit and watch films, and then suddenly the house was empty.” Relative of TB patient, Brazil (BR01).

#### Mixed effects of COVID-19

Some TB patients observed that the COVID-19 pandemic normalized the idea of infection prevention, with mask wearing becoming common. Also, TB patients were able to hide their diagnosis more easily with social distancing measures. There was also less fear that they could infect the wider community. However, access to healthcare and medication was compromised with restrictions to movement and hospitals not accepting admissions for other conditions. Patients were fearful of ‘catching’ COVID-19 given their impaired respiratory health and existing co-morbidities, such as HIV and diabetes. Some respondents who were coming to the end of their isolation and anticipating greater freedoms and a return to a more normal life then faced COVID-19 restrictions.

“During the pandemic I was unable to go to the hospital for my regular follow-ups and prescription renewal, and so because of that my condition worsened, and I eventually ended up infecting my family.” CN08.

## Discussion

Assuming that efficacious treatment is provided, TB is curable. However, outcomes are often sub-optimal. This study aimed to explore common themes in the experiences of TB patients and their families in the five BRICS countries from diagnosis to completion of treatment. Using consistent methodology, economic hardship, stigma, TB-HIV co-infection, disruption to family life, and the mixed effects of COVID-19 were identified as themes encompassing the challenges facing TB patients across the five BRICS countries (Fig. 5). These factors, therefore, appear to be independent of the country setting. Further research should investigate the degree to which these factors and are potentially mutable by targeting systemic changes in healthcare and social provision and providing attention to patients’ individual needs.

Economic hardship was reported across all countries. TB is associated with economic vulnerability but can also drive families into poverty through loss of income, the costs of transportation and food supplements, and associated medical expenses [29–36]. Programs providing social protection to TB patients have been linked to improved outcomes and the increased uptake of preventive therapy but must be easily accessible [29, 37, 38]. Improvement of TB services can also reduce the number of families facing financial hardship [39]. Even though most healthcare systems in our study provided TB drugs free of charge, to be effective, treatment should encompass the wider economic impacts that patients experience. Despite various approaches, patients from all of the countries surveyed found themselves struggling financially and a more holistic approach to patient support is needed.

Stigma attached to TB is culturally distinct, but stems from a lack of awareness of TB and the persistence of stereotypes [40, 41]. For example, in Russia, an association with prisons and poverty has persisted, despite TB affecting all sectors of society [42]. Stigma was most acutely felt in China, and a recent study described psychological distress in nearly two-thirds of TB patients, associated with a high experienced stigma [43]. In our study, some patients did not even disclose their diagnosis to close family. In newly diagnosed Chinese TB patients, non-disclosure of their TB status magnified patient-perceived stigma and was associated with depression – a risk factor for non-adherence [44, 45]. Social support and doctor–patient communication appeared key factors for reducing TB-related stigma in China [46]. Also, educational approaches to raise awareness of TB diagnosis and treatment among the public are needed, particularly focused on those with low educational levels and more rural communities [40, 47, 48].

The association between TB and HIV is well documented. However, the impact on patients is less well understood. In this study, PLWH were more aware of TB and were more likely to seek care early and be diagnosed quickly. This is in contrast to a study in Thailand where PLWH had low TB awareness and attributed their early symptoms to AIDS, resulting in delayed TB diagnosis [49]. This emphasizes the importance of raising TB awareness in PLWH. In South Africa, TB and HIV services are often co-located and integrated [50]. However, a detailed analysis in South Africa of the challenges faced by PLWH who had MDR-TB highlighted similar issues to those described here for all TB patients, such as fear, stigma, dissociation from family and social networks, poor provider support, drug adverse events, and financial insecurity [51]. Also, patients tended to prioritize adherence to anti-retroviral therapy versus TB therapy because it was less challenging in terms of pill burden and adverse effects [52]. Until less demanding treatment regimens are available, targeted support to address the challenges of adherence in patients co-infected with TB-HIV is necessary.

The respondents in this study described a severely disrupted home life following a TB diagnosis. Patients were isolated and often infirm, and the economic and care responsibilities for family members were considerable. Families also suffered socially, being isolated or shunned by friends and the wider family. In many cases, it was family members who ensured adherence to medication, and social and family support for patients has been previously shown as a key factor in therapy adherence [41, 53, 54]. Despite this, the impact of the TB diagnosis on the family and how family members can best be supported has been rarely investigated [47], and we identify this as an important area for further research.

The COVID-19 epidemic has disrupted healthcare access globally [55]. In our study, TB patients reported drug shortages and restrictions to services during the period. TB patients also expressed concern regarding the consequences of contracting COVID-19. Similarly, a recent study in Brazil reported that TB patients were fearful of attending medical appointments [56]. TB patients do appear to be at greater risk of death or poor outcome with COVID-19 [57], and should therefore socially isolate or ‘shield’ [58]. TB patients did feel less stigmatized as social distancing and infection control measures were deployed for COVID-19. However, the interruption of treatment, with the risk of therapy failure, selection of MDR-TB, and increased transmissibility is a major threat to TB patients and their close contacts [59].

This study has several limitations. Although participants were identified through a variety of channels and a range of socioeconomic groups were sampled, this was not a randomized sample and we acknowledge that both marginalized and privileged groups may not engage in this kind of research. Also, there were no data on whether susceptibility testing was conducted following the TB diagnosis, so the appropriateness of therapy could not be assessed. Neither did we examine the differences between patients’ experiences of drug-susceptible versus MDR-TB; patients were not consistently aware of the difference and most patients were receiving or had recently completed first-line therapy. The patient pathway was not integrated into the thematic analysis but analysed separately in terms of the systemic challenges that patients face. This was because the complexity of the pathway did not map onto the themes in a meaningful way. For example, patients experienced economic hardship, stigma, and disruption to family life at most stages in the patient pathway, whereas TB-HIV co-infection had an important effect on the speed of diagnosis. Thus, patient pathway was examined systematically and separately to the thematic analysis which focused on the emotional, socio-economic and practical impacts of TB on patients’ daily lives. The analysis methods sought to remain impartial with repeated reviews by multiple analysts to reach consensus. However, the analysts were all based in the UK and we recognize that the cultural subtleties of some of the patients’ experiences may not have been fully appreciated.

## Conclusions

In our study, TB patients’ perceptions and needs were expressed in their own words, from within their home environment, in confidence, to interviewers who were not involved in their healthcare. Most had struggled to adjust to their diagnosis, had poor access to information, lacked support from healthcare workers, were under significant financial pressure, and were highly conscious of stigma and the burden TB placed on their families.

Our findings highlight that much work still needs to be done before the goal of ending TB can be achieved. Structural changes require simplification of the TB patient pathway, reliable access to services, and the alleviation of financial pressures. Health education for patients, their families, healthcare providers and the public to increase awareness of TB symptoms and diagnosis, to encourage adherence, and to reduce stigma around the disease is needed. Importantly, TB patients do better with strong family and social networks to sustain them, and a greater understanding of how these can be better supported at the level of the individual patient throughout the TB treatment journey requires further investigation.

Despite the different cultural, political, and healthcare settings across the BRICS countries, TB patients faced very similar challenges. This commonality would not necessarily have been expected. It suggests that these factors are not only a product of the healthcare provision in the countries or the social, economic, and cultural pressures that patients face, but reflect an overarching insufficiency in the treatment of TB. The efficient delivery of comprehensive individualized care and support would certainly mitigate the negative impacts of TB on patients. However, these issues will likely not be fully resolved until treatment options are available that rapidly cure TB and prevent onward transmission.

## Supplementary Information

**Additional file 1.** Consent forms.

**Additional file 2.** Screening questions.

**Additional file 3.** Health questionnaire and interview guide.

## Data Availability

All relevant data are included in this publication. Recorded interviews will not be made available in order to maintain patient confidentiality. However, anonymised transcripts are available on reasonable request to the authors for ten years following study completion. For data requests please contact the corresponding author at juliet.x.addo@gsk.com.

## Abbreviations

COVID-19: Coronavirus disease of 2019 (severe acute respiratory syndrome coronavirus 2)
HIV: Human immunodeficiency virus
MDR-TB: Multidrug-resistant tuberculosis
PLWH: People living with HIV
TB: Tuberculosis
