# Supplementary material for: Living with tuberculosis: a qualitative study of patients’ experiences with disease and treatment
Source: BMC Public Health. 2022 Sep 10;22:1717. doi: 10.1186/s12889-022-14115-7 (PMC9462890; doi:10.1186/s12889-022-14115-7)
Supplement: Supplementary file 1 — Additional file 1. Consent forms. [file 12889_2022_14115_MOESM1_ESM.pdf]

PARTICIPANT CONSENT

INTRODUCTION

We are *<insert name of agency>* recruiting for a market research study on behalf of Adelphi Research, an independent market research agency acting on behalf of a pharmaceutical company.

The purpose of the research is to understand your personal experience of living with tuberculosis (TB). We would like to understand your journey with TB from initial symptoms to the present day. We will explore how your TB has been managed, the treatments received, how it affects you in everyday life and what are your needs and expectations for optimal management of your condition. We will also be interested to hear the perspectives of others (e.g. family members) on life with TB, either directly from them if you are comfortable with this, or indirectly through you.

The output of this research will be used by the sponsoring pharmaceutical company in the development of new medicines to ensure that TB patients' needs, experiences, opinions, beliefs and lifestyles are fully considered and communicated. The research is not intended to be promotional in any way.

The research will take the form of either:

- A 60 minute video-streamed interview
    - *This option will include a 60 minute conversation with one of our friendly trained moderators, through a webcam.*

OR

- A 60 minute telephone interview followed by a 45 minute follow up video task
    - *This option will include a 60 minute telephone conversation with one of our friendly trained moderators, followed by an additional 45 minute task which will require you to create 4 short videos on your mobile phone, in your own time.*
    - *For this option, both tasks must be completed to receive the full remuneration*
- A 5 minute questionnaire will take place prior to either form of interview.

You will be compensated for your time with a remuneration of *<insert from incentives table below – recruiter to communicate variation according to task/ additional household members taking part>*

The research will be conducted in accordance with all national data protection laws and relevant industry guidelines that are responsible for regulating research like this, including ESOMAR (European Society for Opinion and Marketing Research), EphMRA (European Pharmaceutical Market Research Association), BHBIA (British Healthcare Business Intelligence Association), and all other relevant national codes of conduct.

ESOMAR: https://www.esomar.org/

EphMRA: https://www.ephmra.org/

BHBIA: https://www.bhbia.org.uk/

We understand that the global coronavirus situation may be placing additional demands on your personal situation. We would like to reassure you that, as with all market research, your participation is voluntary. We understand that the situation is constantly evolving, and that you may no longer be able to participate in this market research. Please be assured that we are aware of these challenges and will be fully flexible to your needs, should you decide to withdraw.

Please review the following statements and confirm your understanding and agreement:

- Any information I provide will be treated as confidential and I will remain anonymous unless I consent otherwise; please note that this consent will be collected throughout the interview process.
- My participation is entirely voluntary, and I have the right to refuse to answer a question or completely withdraw from the research at any time.
- My responses will be used for analysis purposes only, unless I consent otherwise, and I will have the opportunity to confirm my consent at the end of the interview, in case I have changed my mind.
- Findings from the interview may be published in an anonymised format.
- Any information presented to me as part of the interview is confidential and should be assumed to be hypothetical, shared solely to explore my reactions.
- My interview will be available to Adelphi staff in either the UK or US via audio/ video recording <*video interview only:* and live streaming>, for analysis and quality control purposes, but procedures are followed to safeguard my personal data that comply with the data protection legislation in the country in which the interview takes place.
- Employees of the sponsoring pharmaceutical client may <view via video streaming / listen in to> my interview and they may be based outside of the European Economic Area (EEA).
- A separate form will be provided to ask me for and record my consent, if the sponsoring client would like to receive a recording of my interview.
- Support personnel may assist during the interview, including <*simultaneous translators/ technical support staff from FocusVision* > but they are contractually bound to protect my confidentiality.
  - I understand that the screening process may involve profiling or segmenting and making decisions based on my answers.
  - I can confirm that by taking part in this research, I am happy that this will not lead to any negative consequences for myself regarding my personal safety.

ADVERSE EVENT REPORTING

Adelphi are required to pass on to the sponsoring client any details of side effects or product complaints relating to their products that are mentioned during the interview. This is to help them learn more about the safety of their medicines.

If this happens, Adelphi will need to collect details and report the side effects or product complaint.

You will be asked whether you consent to Adelphi passing your details to the company's drug safety department for them to follow up, but you may choose to remain anonymous.

DATA PROTECTION

YOUR RIGHTS UNDER DATA PRIVACY LAWS

As Adelphi Research is based within Europe, your rights under European data protection legislation still apply, even if you are personally based elsewhere.

Under data protection legislation when your personal information is processed, you have certain rights. Please see the recruitment agency's and Adelphi Research's privacy policy for full details on how to exercise these rights.

Adelphi Research only hold your personal data for the purpose of your participation in the market research study. Your personal data will be deleted or destroyed at the end of the project. If you make a request to Adelphi after the end of the project to understand if they are storing any personal data related to you (also known as a subject access request), they would be unable to identify any information attributed to you. You would need to request the information from the recruitment agency.

Are you happy to participate with this interview on this basis? (*If NO, thank and close)*

RECRUITMENT AGREEMENT

In advance of the interview, you will receive a recruitment agreement which you will need to return to *<insert agency>* stating that you have agreed to participate in this research.

RE-CONTACT

We may want to further explore your responses to some of the questions answered. Would you be prepared to be re-contacted by *<insert agency>* for the purpose of hearing more about some of the answers that you my have given? **(*Note response).***

CONFIRMATION OF ADDITIONAL RECORDING CONSENT

Can you confirm that you would be happy for your interview/ video task recording to be used for the following purposes? *Note: This will also be asked following the interview, where you have the opportunity to change or withdraw your response.*

a) Shared with the sponsoring company's market research, Research & Development and market access departments for market research purposes/analysis.
b) Shared with the sponsoring company's market research, Research & Development and market access departments for first hand insight.
c) Shared with the sponsoring company's market research, Research & Development and market access departments for internal training purposes.
d) Shared with the sponsoring company's partner agencies, for example communications or advertising agencies, for internal use of the development of communication materials.
e) Published in an anonymised format in medical journals aimed at healthcare professionals, or medical conferences attended by healthcare professionals.
f) Shared externally in a non-anonymised format in medical journals aimed at healthcare professionals, or external scientific or medical conferences attended by healthcare professionals; a separate consent form will be provided for this.
g) Shared externally in a non-anonymised format on the sponsoring company's website and in communications to provide medical education and insight; a separate consent will be provided for this.

CONTACT AND PRIVACY POLICY DETAILS

Please provide the respondent with the following:

**Recruitment agency:** *<contact details of the recruitment agency (email, telephone number and address)>*
**Recruitment agency's privacy policy:** *<insert link>*

You can:
contact Adelphi Research at [redacted].
find out more about Adelphi Research at www.adelphigroup.com/marketresearch
read Adelphi Research's Privacy Policy at http://www.adelphigroup.com/privacypolicy.pdf

I consent to participate with the interview on the basis of the statements above.

☐ Yes, I agree **[CONTINUE]**

☐ No, I do not agree **[TERMINATE]**

CONSENT FORM – ADDITIONAL ADULT RESPONDENTS

<Note to recruiter: Form to be used following recruitment for consent of additional respondents over 14 years old>

STUDY INFORMATION

<Insert name of agency> are recruiting for a market research study on behalf of Adelphi Research, an independent market research agency acting on behalf of a pharmaceutical company.

WHAT IS THE PURPOSE OF THE RESEARCH?

The main purpose of this market research study is to understand the experience of your family or household member of living with tuberculosis (TB). We would like to understand their journey of living with TB, from initial symptoms to the present day. This includes how their TB has been managed, the treatments received, how it affects everyday life and what needs and expectations exist for optimal management.

As part of this, we are also interested in gathering the perspectives of other important people involved in this journey such as yourself, if you are comfortable with this. During the discussion that has been scheduled with your family or household member, we would like to invite you to contribute to the conversation at various points. The objective of this is to gain a fuller picture of the experience of living with TB, by gaining input from others.

The output of this research will be used by the sponsoring pharmaceutical company in the development of new medicines to ensure that TB patients' needs, experiences, opinions, beliefs and lifestyles are fully considered. The research is not intended to be promotional in any way.

WHAT WILL HAPPEN?

The research will take the form of *<select based on what has been agreed with the primary respondent>:*

- A 60 minute video-streamed interview;
    - *This option will include a 60 minute conversation with one of our friendly trained moderators, through a webcam*

OR

- A 60 minute telephone interview followed by a 45 minute follow up video task.
    - This option will include a 60 minute telephone conversation with one of our friendly trained moderators, followed by an additional 45 minute task which will require you to create 4 short videos on your mobile phone, in your own time
    - For this option, both tasks must be completed to receive the remuneration
- A 5 minute questionnaire will be completed prior to either form of interview.

The research will be conducted in accordance with all national data protection laws and relevant industry guidelines that are responsible for regulating research like this, including ESOMAR (European Society for Opinion and Marketing Research), EphMRA (European Pharmaceutical Market Research Association), BHBIA (British Healthcare Business Intelligence Association), and all other relevant national codes of conduct.

ESOMAR: https://www.esomar.org/

EphMRA: https://www.ephmra.org/

BHBIA: https://www.bhbia.org.uk/

To be included in the study, you must consent to this. Therefore, if you are happy to do so, we ask you to review and agree to the following statements prior to participating in the interview:

Please note that consent is required for each individual that may be included in this either actively participating or in the background, including other adults or children.

- Any information I provide will be treated as confidential and I will remain anonymous unless I consent otherwise.
- My participation is entirely voluntary, and I have the right to refuse to answer a question or completely withdraw from the research at any time.
- My responses will be used for analysis purposes only, unless I consent otherwise, and I will have the opportunity to confirm my consent at the end of the interview, in case I have changed my mind.
- Findings from the interview may be published in an anonymised format.
- My interview will be available to Adelphi staff in either the UK or US via audio/ video recording <*video interview only:* and live streaming>, for analysis and quality control purposes, but procedures are followed to safeguard my personal data that comply with the data protection legislation in the country in which the interview takes place.
- Employees of the sponsoring pharmaceutical client may <view via video streaming / listen in to> my interview and they may be based outside of the European Economic Area (EEA).
- Support personnel may assist during the interview, including *<simultaneous translators/ technical support staff from FocusVision/ Living Lens>* but they are contractually bound to protect my confidentiality.

CONFIRMATION OF ADDITIONAL RECORDING CONSENT

Can you confirm that you would be happy for interview/ video task recordings that you are included in to be used for the following purposes?

Note: This will also be asked following the interview, where you have the opportunity to change or withdraw your response.

a) Shared with the sponsoring company's market research, Research & Development and market access departments for market research purposes/analysis.
b) Shared with the sponsoring company's market research, Research & Development and market access departments for first hand insight.
c) Shared with the sponsoring company's market research, Research & Development and market access departments for internal training purposes.
d) Shared with the sponsoring company's partner agencies, for example communications or advertising agencies, for internal use of the development of communication materials.
e) Published in an anonymised format in medical journals aimed at healthcare professionals, or medical conferences attended by healthcare professionals.
f) Shared externally in a non-anonymised formant in medical journals aimed at healthcare professionals, or external scientific or medical conferences attended by healthcare professionals; a separate consent form will be provided for this
g) Shared externallyin a non-anonymised format on the sponsoring company's website to provide medical education and insight; a separate consent will be provided for this

ADVERSE EVENT REPORTING

This research has been commissioned by a pharmaceutical company that manufactures medicines. It is a legal requirement that the company keep records of any side effects or complaints that people may have about their medicines. We must assist the company in meeting its legal obligations.

Therefore, if, during the interview, you make any reference to a side effect or complaint about any of their medicines, we will let the company know about this even if it has already been reported by you or your doctor, directly to the company or the regulatory authorities. Are you happy to continue on this basis?

YOUR RIGHTS UNDER EUROPEAN DATA PROTECTION LEGISLATION

- As Adelphi Research is based within Europe, your rights under European data protection legislation still apply, even if you are personally based elsewhere.
- Under European data protection legislation when your personal information is processed, you have certain rights. These rights include, but are not limited to: you being able to request a copy of the information which organisations hold on you; a right (in certain circumstances) to object to processing; to withdraw your consent. Please see the recruitment agency's and Adelphi Research's privacy policy for full details on how to exercise these rights.
- Adelphi Research only hold your personal data for the purpose of your participation in the market research study. Your personal data will be deleted or destroyed at the end of the project. If you made a request to Adelphi after the end of the project to understand if they are storing any personal data related to you (also known as a subject access request), we would be unable to identify any information attributed to you. You would need to request the information from the recruitment agency.

I have read, understand and accept the statements contained in this ‘Consent to Participate’ form and consent to participate with the interview on the basis of these statements.

**Consent given** (please tick)	Yes	
**Respondent Signature**	**Name:** (please print)	

In case of further queries, to access, modify or delete your personal data or if you wish to withdraw your consent at any time please contact:
Adelphi Research at: [redacted] .
find out more about Adelphi Research at www.adelphigroup.com/marketresearch
read Adelphi Research’s Privacy Policy at: http://www.adelphigroup.com/privacypolicy.pdf .

The respondent must receive a copy of this form.

If the respondent is not available to sign i.e. telephone/web cam interviews

Consent via email	Consent via telephone
Please email back with the following text:<br><br>*I have read, understand and accept the statements contained in the ‘Consent to Participate’ form and consent to participate with the interview on the basis of these statements <Name>.*	Can you please confirm that you understand and accept the statements that I have just read out from the ‘Consent to Participate’ form and you are happy to proceed with the market research interview on the basis of these statements?<br><br>Interviewer to record respondent’s answer on recording and below:<br><br>☐ Yes, I agree **[CONTINUE]**<br><br>☐ No, I do not agree **[TERMINATE]**

CONSENT FORM – ADDITIONAL CHILD RESPONDENTS

Please ask an adult to help you if there is anything you don't understand.

<Note to recruiter: Form to be used following recruitment for consent of additional respondents 14 years old and under>

STUDY INFORMATION

<*Insert name of agency*> are looking for people to take part in a market research study for Adelphi Research. Adelphi Research is an independent company working for a business that makes medical treatments.

WHAT IS THE PURPOSE OF THE RESEARCH?

The reason we are doing this research is to understand what it is like for a member of your family to live with a condition called tuberculosis (TB).

As part of this, we would like to hear from important people like you, if you are happy to do so. During the conversation with your adult, we would love to hear from you about what your experience has been like.

You are free to say as much or as little as you want, and you are free to change your mind if you decide you don't want to join the discussion. Your adult will always be there during the conversation.

WHAT WILL HAPPEN?

An adult in your house has agreed to take part in *<select based on what has been agreed with the primary respondent>:*

- A 60 minute video call with our friendly team
- A 45 minute task which involves the adult taking videos on a smart phone

It's really important that we protect your information. We follow all laws that are in place to make sure your data is kept safe – this includes any set out by groups responsible for making sure we are keeping to the rules, such as ESOMAR (European Society for Opinion and Marketing Research), EphMRA (European Pharmaceutical Market Research Association), and BHBIA (British Healthcare Business Intelligence Association). We have provided more information to the adults taking part in this on how we will use the information we will collect.

ESOMAR: https://www.esomar.org/

EphMRA: https://www.ephmra.org/

BHBIA: https://www.bhbia.org.uk/

If you would like to take part in this study, we need you to confirm that you are happy to do so. It's completely fine to say no – the choice is yours.

If you are happy to take part, you must agree to the points below:

Please ask an adult to help you if there is anything you don't understand; both you and a responsible adult must agree for you to be allowed to take part.

Note to adults: Please note that consent is required for each individual that may be included in this either actively participating or in the background, including other adults or children.

- Anything I say will be kept private and I will remain anonymous unless I agree otherwise.

- My participation is entirely my choice, and I don't have to answer any questions I don't want to.
- My responses will be used for analysis purposes only, unless I consent otherwise, and I will have the opportunity to confirm my consent at the end of the interview, in case I have changed my mind.
- Findings from the interview may be published (shared with the public) anonymously.
- The audio or video recording of the interview will be available to Adelphi staff in either the UK or US <*video interview only:* and live streaming>, to understand the results, but the law in my country is followed to make sure that my information is kept safe.
- People who work for the company that have paid for this research may <view via video streaming / listen in to> my interview and they may be based outside of the European Economic Area (EEA).
- There may be other people helping to run this interview, including <*simultaneous translators/ technical support staff from FocusVision/ Living Lens*> but it is against the law for them to share any information about me

CONFIRMATION OF ADDITIONAL RECORDING CONSENT

Please ask an adult to help you if there is anything you don't understand; both you and your parent/ guardian must agree for you to be allowed to take part

Can you confirm that you would be happy for interview/ video task recordings that you are included in to be used for the following reasons? Again, the choice is up to you.

Note: This will also be asked following the interview, where you have the opportunity to change your mind

h) Shared with the sponsoring company's market research, Research & Development and market access departments for market research purposes/analysis.
i) Shared with the sponsoring company's market research, Research & Development and market access departments for first hand insight.
j) Shared with the sponsoring company's market research, Research & Development and market access departments for internal training purposes.
k) Shared with the sponsoring company's partner agencies, for example communications or advertising agencies, for internal use of the development of communication materials.
l) Published in an anonymised format in medical journals aimed at healthcare professionals, or medical conferences attended by healthcare professionals.
m) Shared externally in a non-anonymised format in medical journals aimed at healthcare professionals, or external scientific or medical conferences attended by healthcare professionals; a separate consent form will be provided for this.
n) Shared externally in a non-anonymised format on the sponsoring company's website to provide medical education and insight; a separate consent will be provided for this.

ADVERSE EVENT REPORTING

This research is run on behalf of a company that makes medicines. The law says that the company must keep records of any problems that people may have about their medicines. We must help with this too.

Therefore, if, during the interview, you mention any problems about any of the company's medicines, we will let the company know about this even if an adult has already done so. Are you still happy to take part?

YOUR RIGHTS UNDER EUROPEAN DATA PROTECTION LEGISLATION

Please ask an adult to help you if there is anything you don't understand

- Under the European law (data protection legislation) when people hold information about you, there are certain things you can ask for. This includes you being able to see a copy of the information which a company has about you; the choice to (in certain circumstances) to not want anything done to your information; the choice to no longer take part. Please talk to an adult who will be able to see our privacy policy for more information.
- We only hold information for the purpose about you as part of this research. Information about you will be deleted or destroyed once this is finished. If an adult responsible for you asked to see the information we have about you after the end of the project, we would be unable to identify any information about you. You would need to talk to an adult who could request the information from the recruitment agency.

I have read, understand and agree to everything in this 'Consent to Participate' form and agree that I am happy to take with the interview on the basis of these statements

**Consent given** (please tick)	Yes	
**Respondent Signature**	**Name:** (please print)	

**Consent given** (please tick)	Yes	
**Parent Signature**	**Name:** (please print)	

Both respondent under 14 years old and a parent/ guardian must consent for the respondent to be able to take part
<Note to recruiter: Amend for local markets differences on child research guidelines where relevant>

In case of further queries, to access, modify or delete your personal data or if you wish to withdraw your consent at any time please contact:
Adelphi Research at: [redacted] .
find out more about Adelphi Research at www.adelphigroup.com/marketresearch
read Adelphi Research's Privacy Policy at: http://www.adelphigroup.com/privacypolicy.pdf .

The respondent must receive a copy of this form.

If the respondent is not available to sign i.e. telephone/web cam interviews

**Consent via email**	**Consent via telephone**
Please email back with the following text:<br><br>*I have read, understand and accept the statements contained in the 'Consent to Participate' form and consent to participate with the interview on the basis of these statements <Name>.*	Can you please confirm that you understand and accept the statements that I have just read out from the 'Consent to Participate' form and you are happy to proceed with the market research interview on the basis of these statements?<br><br>Interviewer to record respondent's answer on recording and below:<br><br>☐ Yes, I agree **[CONTINUE]**<br><br>☐ No, I do not agree **[TERMINATE]**
