# Supplementary material for: Living with tuberculosis: a qualitative study of patients’ experiences with disease and treatment
Source: BMC Public Health. 2022 Sep 10;22:1717. doi: 10.1186/s12889-022-14115-7 (PMC9462890; doi:10.1186/s12889-022-14115-7)
Supplement: Supplementary file 2 — Additional file 2. Screening questions. [file 12889_2022_14115_MOESM2_ESM.pdf]

SCREENING QUESTIONS

S1.	Are you, or any member of your household, employed by the pharmaceutical industry on a full-time or part time, or is any member of your household employed by a communication or advertising industry related to pharmaceutical products?<br><br>Yes .... 1 CLOSE<br>No 2
S2.	Are you currently suffering, or have recently recovered, from tuberculosis as confirmed through diagnosis by a healthcare professional or healthcare worker?<br>Yes .... 1<br>No .... 2 CLOSE<br><br>Date of diagnosis<br>___________________ (MM/YY)<br><br>MUST HAVE CONFIRMED DIAGNOSIS OF TB 2017 OR LATER, OTHERWISE CLOSE
S3.	A. What stage of treatment are you currently at for your tuberculosis?<br>Not started treatment .... 1 CLOSE<br>Currently receiving 1st treatment .... 2<br>Currently receiving 2nd treatment (or later), as my previous treatment did not work as well as expected 3<br>Currently receiving 2nd treatment (or later), due to being re-infected .... 4<br>Currently receiving 2nd treatment (or later), as I suffered side effects from my previous treatment 5<br>Have previously received treatment but currently on a break .... 6<br>Treatment programme completed .... 7<br>IF CODE 3 SELECTED, RECRUIT AS MDR/ XDR; PHYSICIAN REFERRALS TO HELP INFORM, WHERE PATIENTS ARE UNAWARE OF STATUS<br>RECRUIT MIX<br><br>B. *IF CURRENTLY TREATED (S3a_2/3/4):* How long have you been on treatment?<br>< 3 months .... 1<br>3-6 months .... 2<br>6-9 months .... 3<br>>9 months 4

	C. *IF TREATMENT PROGRAMME COMPLETED (S3a_6):* How long have you been treatment-free?<br>< 3 months .... 1<br>3-6 months .... 2<br>7-12 months .... 3<br>>12 months .... 4 CLOSE<br>MUST HAVE COMPLETED TREATMENT IN THE LAST 12 MONTHS

Recruiter read out:

As part of this research, we would like to speak to people such as yourself who are representative of different types of patients suffering from TB. This may include different characteristics such as your gender, the area in which you live, any other health conditions you suffer from and your feelings towards having TB.

Therefore, the next few questions are to understand this.

S4.	RECRUITER RECORD GENDER<br>Male .... 1<br>Female .... 2<br>Non-Binary .... 3<br>REFER TO QUOTA TABLE
S5.	To what extent do you agree or disagree with the following statements that describe how you may feel about TB?<br>*Please answer on a 5 point scale where 1= strongly disagree and 5 = strongly agree*

	Strongly Disagree ***1***	***2***	***Neutral*** ***3***	***4***	***Strongly Agree*** ***5***
I feel hurt how others react to knowing I have TB.					
I lose friends when I share with them that I have TB.					
I am afraid to tell my family that I have TB.					
I am afraid to tell those outside my family that I have TB.					
I choose carefully who I tell about having TB					

RECRUIT MIN n=1 SCORING >3 FOR AT LEAST 2 STATEMENTS

S6.	Do you suffer from any of the following conditions?<br>Diabetes .... 1<br>HIV .... 2<br>Cardiovascular disease .... 3<br>Depression .... 4<br>Other .... 5<br>None of the above .... 6<br>REFER TO QUOTA TABLE
S7.	*CHINA ONLY:*<br>Do you currently smoke?<br>Yes .... 1<br>No .... 2<br>MUST RECRUIT AT LEAST n=1 CURRENT SMOKER
S8.	*RUSSIA ONLY:*<br>Do you currently suffer from, or have you ever suffered from alcohol dependence?<br>Yes .... 1<br>No .... 2<br>MUST RECRUIT AT LEAST n=1 WITH CURRENT OR PREVIOUS ALCOHOL DEPENDENCE; REFER TO QUOTA TABLE

S9.	A. How many children (<14 years old) live in your household?<br>0 .... 1<br>1 .... 2<br>2 .... 3<br>3 or more .... 4<br><br>*B. <If 1 or more children in household>*<br>Of the children in your household, do any fall under the following categories:<br>Currently suffering from TB, as diagnosed by a HCP or health worker .... 1<br>Currently receiving preventative TB treatment .... 2<br>None of the above .... 3<br><br>MUST RECRUIT AT LEAST n=2 WITH CHILDREN SUFFERING FROM OR TAKING PREVENTATIVE TREATMENT FOR TB; REFER TO QUOTA TABLE
S10.	What type of healthcare coverage do you have access to?<br>Private .... 1<br>Public .... 2<br>RECRUIT MIX OF COVERAGE
S11.	Socio-economic status<br>*<insert country specific questions from appendix>*<br>RECRUIT MIX
S12.	ALL EXCEPT SA<br>In what type of area is your main place of residence? If you are currently staying in a hospital, please consider the place where you would normally live<br>Urban .... 1<br>*Inner city dwelling*<br>Suburban .... 2<br>*Outer city dwelling, on outskirts of city*<br>Rural .... 3<br>*Dwelling outside of a town or city*<br>RECRUIT MIX; REFER TO QUOTA TABLE

S13.	In what region are you based?<br><br>*<insert local region list>*<br><br>RECRUIT MIX OF REGIONS
S14.	Do you have access to the following?<br>• A smart phone in which you are able to record/ upload videos<br>• A webcam to be able to take part in a video-streamed interview<br><br>RECORD RESPONSE<br><br>NOTE: RESPONDENTS SHOULD BE PROVIDED WITH ACCESS TO TECHNOLOGY TO COMPLETE INTERVIEW IF REQUIRED; LIAISE WITH RESPONDENT TO ORGANISE ACCESS WHERE POSSIBLE
S15.	We are also interested in gaining the views of others close to you, for example members of your family or household, within the interview/ task to further understand your experience of living with TB.<br><br>a) Would you be willing to include others close to you, such as your household, within the interview and/ or video tasks?<br><br>Yes..1<br>No ....2<br><br>IF YES:<br><br>b) How many additional people will participate?<br><br>______________________<br><br>c) Of these additional people, will any participants be under 14 years old (at the time of interview)?<br><br>Yes..1<br>No ....2<br><br>RECORD RESPONSE;<br><br>IF YES, SHARE ADDITIONAL CONSENT FORM TO ALL ADDITIONAL RESPONDENTS;<br><br>IF CHILDREN INCLUDED, ENSURE EXPLICIT CONSENT GAINED FROM BOTH CHILD RESPONDENT AND THEIR PARENT

Socio-Economic Status – Country Specific Questions

TO BE INSERTED AT S12 ACCORDING TO COUNTRY

INDIA	What is your total household income per annum?<br>< ₹ 2,50,000 .... 1<br>₹ 2,50,001-₹ 5,00,000 .... 2<br>₹ 5,00,001-₹ 7,50,000 .... 3<br>₹ 7,50,001-₹ 10,00,000 .... 4<br>₹ 10,00,001-₹ 12,00,000 .... 5<br>₹ 12,00,001-₹ 15,00,000 .... 6<br>₹ 15,00,001-₹ 18,00,000 .... 7<br>₹ 18,00,001-₹ 20,00,000 .... 8<br>> ₹ 20,00,000 .... 9<br>RECRUIT MIX, AS PER LOCAL SES SCORING SYSTEM
	Postgraduate/ Master's degree/ Doctorate .... 1<br>Graduate/ Bachelor's degree .... 2<br>Attended university but did not complete .... 3<br>Completed Diploma/ Technical Course .... 4<br>Intermediate/ Up to grade 12 .... 5<br>High School/ Up to grade 10 .... 6<br>Primary (Up to grade 5) 7

RUSSIA	Which statement more accurately describes the financial situation of your family?< 10,000 rubles....1<br>Not enough money for food....1<br>Enough money for food but buying clothes is difficult ....2<br>Enough money for food and clothes but not enough for household appliances ....3<br>Enough money to buy household appliances, but not enough to buy a new car ....4<br>Enough money for all purchases except real estate....5<br>No financial difficulties ....6<br>RECRUIT MIX (LIKELY CODES 1-4 ONLY)
	What is the highest level of education that you have completed?<br>Incomplete secondary education (less than 11 classes) ....1<br>Secondary general education (11 classes of the school)....2<br>Specialized secondary (professional) education ....3<br>Unfinished higher (three years or more)....4<br>Higher education (bachelor, specialist, master)....5<br>Academic degree (candidate of sciences, doctor of sciences) 6

CHINA	What is your total household income per annum?<br><RMB50,000 … 1<br>RMB50,000-RMB79,999. … 2<br>RMB80,000-RMB159,999 … 3<br>RMB160,000-RMB239,999 … 4<br>RMB240,000-RMB319,999 … 5<br>RMB320,000-RMB399,999 … 6<br>RMB400,000-RMB499,999 … 7<br>RMB500,000-RMB599,999 … 8<br>> RMB600,000 … 9<br>RECRUIT MIX (LIKELY CODES 2-6)
	What is the highest level of education that you have completed?<br>Master’s degree/ Doctorate … 1<br>Bachelor’s degree … 2<br>Higher education … 3<br>High school diploma or equivalent … 4<br>Less than high school diploma … 5

7

SOUTH AFRICA

RECRUITER: please identify LSM category using the following flow diagram and then confirm using the income brackets below

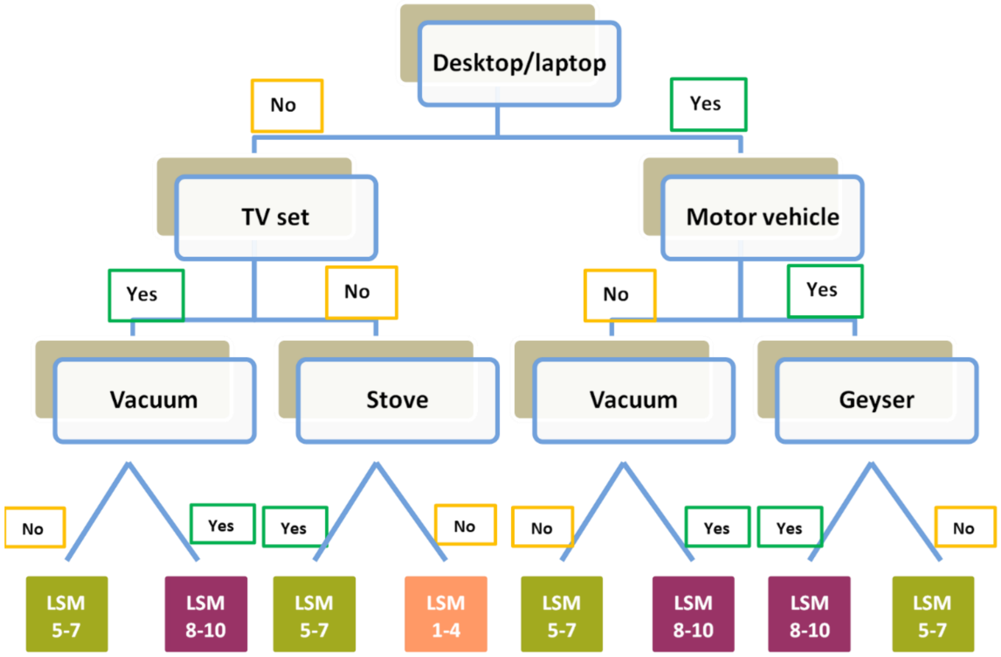

What is your total household income per month?

		LSM:
Up to R3 330	1	1-4
R3 331 – R5 115	2	5
R5 116 – R8 615	3	6
R8 616 – R12 650	4	
R12 651 – 20 030	5	8
R20 031 – R25 180	6	9
R25 181+	7	10

RECRUIT MIX OF LIVING STANDARD MEASUREMENT (LSM) SCORES

BRAZIL	I will now ask you a few questions about items in your household, for economic classification purposes. All electrical/ electronic items I will mention must be in working condition, including any items that are stored. In case an item is not working, please include It only if you intend to have it fixed or replaced within the next six months.<br><br>INSTRUCTION: All items must be asked by the interviewer and answered by the respondent<br><br>*In your household, there is/ are ______*

**The water used in this household comes from…?**	
1	The utility company's distribution system
2	Well or spring
3	Other

**Considering the stretch of street your household is at, you would say your street is…:**	
1	Asphalt/Paved
2	Dirt/Gravel

		NUMBER IN HOUSEHOLD			
COMFORT ITEMS	NOT OWNED	1	2	3	4+
Number of passenger cars exclusively for personal (i.e., not professional) use					
Number of monthly servants, including only those who work at least five days a week					
Number of washing machines, excluding tub washing machines					
Number of bathrooms, restrooms, powder rooms					
DVD Players, including any device that plays DVDs, and excluding Car DVD Players					
Number of refrigerators					
Number of stand-alone freezers, or freezers in two-door refrigerators					
Number of personal computers, including desktop computers, laptops, notebooks and netbooks, and excluding tablets, palmtops or smartphones					
Number of dishwashers					
Number of microwave ovens					
Number of motorcycles, not considering those used exclusively for professional activities					
Number of clothes dryers, including washers-and-dryers					

What is the householder's education?

Analfabeto / Fundamental I incompleto	No schooling / Incomplete Elementary School
Fundamental I completo / Fundamental II incompleto	Elementary School Diploma / Incomplete Junior High School
Fundamental completo/Médio incompleto	Junior High School Diploma / Incomplete High School
Médio completo/Superior incompleto	High School Diploma / Incomplete Higher Education
Superior completo	Higher Education Degree

POINTS SYSTEM

	Number of Items				
	0	1	2	3	4 or +
Bathroom(s)	0	3	7	10	14
Domestic servant(s)	0	3	7	10	13
Automobile(s)	0	3	5	8	11
Personal computers(s)	0	3	6	8	11
Dishwasher(s)	0	3	6	6	6
Refrigerator(s)	0	2	3	5	5
Freezer(s)	0	2	4	6	6
Washing machine(s)	0	2	4	6	6
DVD player(s)	0	1	3	4	6
Microwave oven(s)	0	2	4	4	4
Motorcycle(s)	0	1	3	3	3
Clothes dryer(s)	0	2	2	2	2

Householder's education	
No schooling / Incomplete Elementary School	0
Elementary School Diploma / Incomplete Junior High School	1
Junior High School Diploma / Incomplete High School	2
High School Diploma / Incomplete Higher Education	4
Higher Education Degree	7

	Public Utility Services	
	No	Yes
Piped water	0	4
Paved street	0	2

	THRESHOLDS<br>A .... 45-100 points<br>B1 .... 38-44 points<br>B2 .... 29-37 points<br>C1 .... 23-28 points<br>C2 .... 17-22 points<br>D-E .... 0-16 points
