# Supplementary material for: Living with tuberculosis: a qualitative study of patients’ experiences with disease and treatment
Source: BMC Public Health. 2022 Sep 10;22:1717. doi: 10.1186/s12889-022-14115-7 (PMC9462890; doi:10.1186/s12889-022-14115-7)
Supplement: Supplementary file 3 — Additional file 3. Health questionnaire and interview guide. [file 12889_2022_14115_MOESM3_ESM.pdf]

HEALTH QUESTIONNAIRE

Please take a few minutes to complete the three questions below, ahead of the telephone discussion we have arranged. If possible, please send your completed questionnaire back to <Insert agency details>.

Please be assured that the information you provide us with will be treated as confidential and will remain anonymous.

If you need any help completing the questionnaire or are unable to send it back, please let us know and we will be very happy to assist.

Many thanks.

**1.**	**During the past four weeks, were you limited in the kind of work or other activities you could do as a result of your physical health?**		
	a) Yes		
	b) No		
**2.**	**If yes: How much of the time were you limited in the kind of work or other activities you could do?**		
	a) All of the time		
	b) Most of the time		
	c) Some of the time		
	d) A little of the time		
	e) None of the time		
**3.**	**For each statement please select the box that applies to you these days:**	True	False
	a) My cough or breathing is embarrassing in public		
	b) My TB is a nuisance to my family, friends or neighbours		
	c) I feel that I am not in control of my TB		
	d) I have become frail or an invalid because of my TB		
	e) My TB has caused economic hardship to me and my family		
	f) My TB has stopped me doing things I would like to do		

INTERVIEW GUIDE

I. Introduction (8 MINUTES)

Objectives: To understand the respondent's life situation and context, and to create rapport and put them at ease

---

I'd like to start our conversation today by getting to know you, before we talk about tuberculosis (TB).

A. Please introduce yourself and tell me:
   1. Your first name (no surname)
   2. Your home life, including family or others you may live with **[For wTDIs, invite respondent to introduce carers where consent gained]**
   3. Your close friends and their role in your life
   4. Any employment
   5. How you like to spend your free time, e.g. any hobbies or interests

B. Thinking about your family members or close friends, if I were to ask them to describe you in three words, what would they say? **[For wTDIs, invite carers to answer directly]**
   1. Why do you think they would choose these words?

C. Now I would like to gain a brief understanding of your health situation
   1. When were you diagnosed with tuberculosis (TB)?
   2. Please tell me about any other health conditions you may have **[refer to screener answers]**
   3. When were any other members of your household diagnosed?

D. What impact, if any, has **Covid-19 (coronavirus)** had on the way in which your TB has been managed?
   1. Have you been tested for Covid-19?
      a) What was the outcome?
   2. Has Covid-19 affected:
      a) Your ability to access medication?
      b) Monitoring or access to appointments?
      c) Anything else?

II. Your TB journey (25 MINUTES)

Objectives: To fully explore the patient journey from initial symptoms to the present day, focusing on what happened, when, who was involved, and challenges faced.

I would now like to hear about your experiences right back from when you started having symptoms, up to the present day. Later in our conversation we will focus specifically on the impact of TB on your life, but for now our focus will be on what happened at each point, at what time, and who was involved.

Initial symptoms and presentation

E. What **symptoms** did you first notice?
   1. When was this?
   2. How were you feeling at this point?

F. What **did you first do** when you noticed these symptoms?
   1. E.g. Look for information, ask family and friends, see a healthcare professional, see a non-healthcare professional (such as traditional healers)

G. What specifically prompted you to **visit a healthcare professional or healthcare worker**?
   1. How long after first noticing symptoms did you see a healthcare professional/ worker?
      a) If any delays: What were the reasons for the delay?
      b) Did you have to travel or take any time off work?
   2. Which healthcare professional/ worker did you visit? **[Probe for whether in the public or private sector]**

H. What happened in this **first consultation**?
   1. How were you feeling at this point?
   2. What was the outcome of the consultation?

I. Were you **referred to any other healthcare professional(s)** at this point?
   1. If so, which type of healthcare professional?
   2. Did you have to wait for an appointment? If yes, how long??
   3. What was your understanding of the reasons for the referral?
   4. What happened when you saw this healthcare professional?

The diagnostic process

J. I would now like to understand the **process of getting a diagnosis** of TB
   1. Which healthcare professionals were involved in this process?

2. What were you told at this point?
    a) Were you aware that you were being tested for TB?
3. What tests can you recall, if any?
    a) What was your understanding of the reason for these tests?
4. How were you feeling at this point, before you got the diagnosis of TB?

K. Now I would like to focus on the **point at which you were given the diagnosis** of TB

1. Who gave you the diagnosis of TB?
2. What, if anything, did you know about TB at this point?
    a) What was your perception of TB?
3. What were you told? **Allow spontaneous response then probe on:**
    a) What TB is
    b) Impact on your life, e.g. family and work
    c) Impact on other conditions
    d) Transmission
    e) Treatment
    f) The need to stay in a hospital or treatment centre
    g) Possible outcomes
4. What education or support were given to you, if any? **Allow spontaneous response then probe on:**
    a) Digital e.g. online, text message
    b) Paper materials, e.g. leaflets?
    c) Any other support
    d) What did these include/ look like?
5. Please describe your feelings at this point of receiving the diagnosis
    a) What were your biggest fears or concerns?
6. How long did it take to get a diagnosis, from the time you first saw a healthcare professional?

L. Did you **tell anyone else** about your diagnosis?

1. Who did you tell?
2. What did they say or do?
3. How did you feel at this point?
4. *If not:* Why not?

Treatment

M. I would now like to talk about the **decision to start treatment**

1. Which healthcare professional did you see at this point?
2. How was the decision made to start treatment?
    a) How much input did you have in treatment choice?
    b) How did you feel about the amount of input you had?
3. What is the name of the treatment(s) you are/were on? [if name not known, probe on mode of administration, frequency, duration etc)
    a) How many tablets/injections
    b) How often do you take them/have the injections?
    c) How long are you expected to continue with this for?
    d) How is this monitored, and by who? *Probe on whether patient is on direct observed therapy (DOTs) i.e. a healthcare worker watches them take the medication*
4. What were you told about treatment?
    a) Benefits?
    b) Drawbacks?
    c) How to take the treatment?
5. How did you feel about starting treatment?
    a) What were your biggest fears or concerns?
6. How long after getting the diagnosis did you start treatment?

N. I would now like to talk about **your experience on this treatment**

1. How do/did you feel?
    a) What is/was good?
    b) What is/was not so good?
2. What is/was your experience of the following aspects of treatment?
    a) Ease of getting your medication (e.g. distance travelled)
    b) Cost of medication
    c) Side effects
    d) Impact on your daily life
3. We know that it can be difficult to take medication exactly as explained by the doctor. Please tell me about any challenges you face/d with taking your medication
    a) Did you speak to the doctor about this?
    b) Were you given any additional support?
    c) How useful was this?
4. How long were you on this treatment for/expect to be on treatment for?

O. Did you **change treatment** at any point? If so:

1. For what reasons was your treatment changed? **Allow spontaneous response then probe on:**

a) Treatment not working as well as it should

(1) What was your understanding of the reasons that treatment was not working?

b) Side effects

(1) Which side effects?

(2) What impact did these have?

c) Difficulties taking the medication

(1) What challenges did you face?

2. **Briefly repeat questions I and J above for subsequent treatment**

Ongoing monitoring [please adapt questions according to the point in the journey the patient is at, and whether treatment is directly observed therapy (DOTs)]

P. Please describe how your **condition was/is being monitored** on an ongoing basis?

1. What type of healthcare professional or other persons monitors/ed your condition?

   a) were family or other members of the community involved? Who?

2. Where did/do you see them (e.g. at home, in a hospital, in a clinic)?

   a) Did/does this change at different points in time? *Probe on differences between DOT and additional monitoring*

   b) If not already mentioned: Did you have to stay in hospital or a treatment centre at any point?

3. How often were/are you monitored?

   a) Did/does this change with different people *[DOT vs. additional monitoring]* at different points in time?

4. What was/is your understanding of the purpose of monitoring? *Probe on differences between DOT and additional monitoring*

1. How did/do you feel about the process of being monitored on an ongoing basis?

2. We know that it can be difficult to keep up with these appointments *<and observations>* over time. Please tell me about any challenges you face/d with your ongoing monitoring. *Probe on differences between DOT and additional monitoring*

   a) Did you speak to the doctor about this?

   b) Were you given any additional support?

   c) How useful was this?

3. How long were you monitored for/do you expect to be monitored for?

   If DOT: How does this direct observed therapy impact you?

   a) What was your experience of this?

   b) What challenges did you face, if any?

   c) What challenges did others involved face, if any

III. Life with TB (25 MINUTES)

Objectives: To understand the impact of living with TB on patients' lives, covering what they think, feel and can do across various aspects of life.

We will now change focus and talk about what living with TB has been like for you. We will discuss the changes you have had to make in your daily life, and the impact this has had on you.

Moderator refer back to answers from one-page health questionnaire and probe on any limitations as appropriate during this section. Please also adapt the questions below as appropriate for those who have suffered from TB previously but are now recovered/recovering.

A. First of all, I would like you to tell me three words to describe **how you feel about living with TB/having lived with TB**

1. Why did you choose these words?
2. **[For wTDIs, invite carers to provide three words also]**

B. Now I would like you to do a short exercise to understand **how you see TB as a condition**. I would like you to imagine that TB comes to life as a person. Please describe what they would be like. **Allow spontaneous response then probe on:**

1. What would their character be like?
2. How would they behave?
3. How would they interact with others?
4. What would you wish to say to them?
5. Why have you described TB in this way?

C. I would like to spend some time hearing about the impact that TB has had on your life. First of all, please tell me the **three biggest challenges** you have had to face due to TB.

1. What is the first biggest challenge? Second? Third?
2. Please explain each of these challenges in as much detail as possible

D. In what ways has living with **TB had an impact** on your life? **Allow respondent to speak freely on their own terms, with minimal interruption before probing**

1. Please describe what a good day with TB looks like?
2. And what does a bad day look like?
3. How does this compare to life before TB?

E. Now I would like to ask about **specific aspects of your life that may have been affected** by TB. We will go through each of these in turn, so that I can understand fully what has changed, the impact this has had, and how it has made you feel. **Cover each of the following areas:**

1. Ability to carry out daily activities
    a) Impact of fatigue
    b) Impact on sleep patterns
    c) Impact of changes to sense of smell or taste
    d) Any adaptations they have made due to changes in their ability to carry out normal, daily activities
2. Family / home life / living arrangements
    a) Any specific adaptations made?
    b) Impact on children in the household
    c) **Refer to screener answers and probe on impact on children also diagnosed with TB or on preventative medication:**
        (1) Please describe your child's experience of living with TB
        (2) What specific challenges has your child faced?
        (3) For wTDIs, invite child to give their perspective also
3. Management of possible transmission
4. Work life / employment
5. Social life / personal relationships
    a) Any experience of stigma from others?
    b) How does this make them feel?
6. Mood / mental health
7. Finances
8. Access to healthcare facilities
    a) Distance travelled to access healthcare
    b) Frequency of impact
    c) Issues of transmission on access to healthcare
    d) Issues of regular monitoring through DOTs (where relevant) **[For wTDIs, invite carers to give their perspective if involved in this]**
9. Impact on other health conditions
    a) **Refer to screener answers and probe on impact on specific conditions**
10. How you have been treated by others **[if not already covered above]**

For each of the above areas, probe on:

1. What changes have you had to make? **[For wTDIs, invite respondent to show any changes on camera where applicable]**
2. What impact has this had on your life?
3. What impact has it had on others around you?
4. How has this made you feel?
5. **[For wTDIs, invite carers to give their perspective on these issues]**

F. I would now like to focus on the **information or support** you may have been provided with about TB at various points

1. What type of information have you received?
    a) In what format?
2. At what points were you offered this information or support?
3. How useful was this information or support?
    a) How easy was it to access this information?
    b) How easy was it to understand the information?
    c) To what extent did it help you to understand TB?
    d) To what extent did it help you to make decisions about your condition?
    e) To what extent did it help you to manage treatment?
    f) What could have been improved?
4. **[For wTDIs, invite carers to give their perspective on information or support offers]**

G. Now I would like to hear more about your **interactions with the healthcare professionals and other healthcare workers** you have seen along the way

Probe on differences between DOTs and other interactions where relevant.

1. How do you feel about the care you have received?
2. How would you describe the relationship with the HCP(s)?
3. To what extent do you feel that your wishes and needs have been considered?
4. What has worked well?
5. What could have been improved?

IV. Wrap-up (2 MINUTES)

Objectives: To bring the conversation to a close on a positive note

We are now coming to the end of our discussion.

A. Let's imagine that someone else who has just been diagnosed with TB asks you for advice on living with TB. What would be your **top three tips** for them?

   1. Why are these things important?

B. This brings us to the end of our conversation. We would like to thank you so much for your time today. We very much appreciate you talking to us and sharing your experiences with TB.

C. **For TDI respondents who are taking part in the mobile ethnography task:** I would like to give you some more information about the second task you will take part in

   1. The next task will involve collecting some video footage via an app on a smart phone, or using the video function on a smart phone
   2. We will set 4 tasks, and will provide full details on what we are looking for in each task
   3. Each task states how long you need to spend on it; this will add up to 45 minutes in total
   4. If you have any difficulties with completing the tasks or would like assistance with technology, please do not hesitate to get in touch

AT THE END OF THE DISCUSSION GUIDE

Naming Client: (at end) The company sponsoring this research is GSK.

Confirmation of consents

Can you confirm that you would be happy for your interview/ video task recording to be used for the following purposes?

a) Shared with the sponsoring company's market research, Research & Development and market access departments for market research purposes/analysis
b) Shared with the sponsoring company's market research, Research & Development and market access departments for first hand insight
c) Shared with the sponsoring company's market research, Research & Development and market access departments for internal training purposes
d) Shared with the sponsoring company's partner agencies, for example communications or advertising agencies, for internal use of the development of communication materials
e) Published in an anonymised format in medical journals aimed at healthcare professionals, or medical conferences attended by healthcare professionals
f) Shared externally in a non-anonymised format in medical journals aimed at healthcare professionals, or external scientific or medical conferences attended by healthcare professionals; a separate consent form will be provided for this.
g) Shared externally in a non-anonymised format on the sponsoring company's website to provide medical education and insight; a separate consent will be provided for this.
